# An appropriate Wnt/β-catenin expression level during the remodeling phase is required for improved bone fracture healing in mice

**DOI:** 10.1038/s41598-017-02705-0

**Published:** 2017-06-02

**Authors:** Quanwei Bao, Sixu Chen, Hao Qin, Jianquan Feng, Huayu Liu, Daocheng Liu, Ang Li, Yue Shen, Yufeng Zhao, Junfeng Li, Zhaowen Zong

**Affiliations:** 1 State Key Laboratory of Trauma, Burn and Combined injury, Department of Trauma Surgery, Daping Hospital, Third Military Medical University, ChongQing, 400042 China; 2 Department of Biomedical Sciences, Baylor College of Dentistry, Texas A&M Health Science Center, Dallas, TX 75246 USA

## Abstract

Accumulating evidence demonstrates that the Wnt/β-catenin signaling pathway plays a dominant role in bone repair. However, the role of Wnt/β-catenin signaling in the remodeling phase during bone fracture healing is currently unknown. In the present study, β-catenin was activated at different levels or deleted in mice at the late stage of fracture healing, and the effects on healing quality were investigated. Deletion of β-catenin disturbed bone remodeling, as confirmed by increased bone resorption and decreased bone formation, and significantly decreased bone strength compared with wildtype mice. In addition, the constitutive activation of β-catenin significantly increased the bone mass and delayed the bone remodeling process, resulting in slightly impaired bone strength. In contrast, a slight activation of β-catenin significantly increased bone formation and slightly hindered bone resorption. These effects lead to improved bone fracture healing quality compared with wildtype mice. In summary, the present study provides the first demonstration showing that Wnt/β-catenin signaling should be maintained at a slightly activated level during the late stage of fracture healing to ensure better bone fracture repair.

## Introduction

Secondary bone healing is the most common type of fracture healing observed in a clinical setting. This process, which is the same in mice and human, consists of the following three closely linked phases: (1) an inflammatory phase involving hematoma formation and the recruitment of mesenchymal stem cells, (2) a reparative phase characterized by the periosteal response, chondrogenesis, formation of a vascularized cartilaginous callus and formation of woven bone via endochondral ossification, and (3) a remodeling phase involving resorption and formation, which shape the callus and restore the bone to its original morphology and quality^1–3^. Fracture healing consists of a variety of molecular and cellular events and is controlled by numerous, complex cellular signaling pathways. Studies in mice and humans have revealed numerous signaling pathways that are potential new therapeutic targets for regenerating new bone, and Wnt/β-catenin signaling is one of the most important of these signaling pathways^1, 3–5^.

The Wnt/β-catenin signaling pathway, also known as the canonical Wnt pathway, inhibits the phosphorylation and degradation of β-catenin, which accumulates in the cytoplasm and translocates into the nucleus, where it binds to transcription factors (ternary complex factor/lymphoid enhancer factor 1, TCF/LEF1) and regulates the expression of a number of targeted genes. The altered expression of targeted genes then exerts their biological effects.

Wnt/β-catenin signaling is evolutionarily conserved and expressed in the main three types of cells in bone tissue, i.e., osteoblasts, osteoclasts and osteocytes. Wnt ligands, including Wnt4, Wnt5a, Wnt5b, Wnt10b, Wnt11 and Wnt13, Wnt receptors, including Fzd1, 2, 4, 5, and 9, the co-receptors Lrp5 and Lrp6, β-catenin, and Wnt target genes, including Runx2 and dentin matrix protein-1, have been demonstrated to be up-regulated in the fracture callus during bone regeneration^3–5^. In addition, the activation of Wnt signaling through the administration of a canonical Wnt agonist or neutralizing antibodies against the canonical Wnt inhibitors dickkopf-1 and sclerostin have been found to improve bone healing in mice^6–14^. The mechanisms underlying the Wnt/β-catenin signaling pathway in favoring fracture healing include attracting mesenchymal stem cells to the injury site, controlling the differentiation of MSCs into osteoblasts instead of chondrocytes, enhancing bone formation, and decreasing bone resorption^6–14^.

More recent findings suggest that Wnt signaling should be precisely regulated in a stage-specific manner to allow complete fracture healing^4, 5, 7, 15^. In an endochondral setting, β-catenin appears to exert different effects at different stages of bone repair. Early in the process, the protein controls the relationship between the numbers of osteoblasts and chondrocytes that arise from pluripotent mesenchymal cells. Thus, both too much and too little β-catenin can be detrimental to bone healing at this stage. Later during the process, β-catenin promotes the differentiation of osteoblasts and enhances their production of the bone matrix; thus, too little β-catenin at this stage impairs healing^4, 7^, whereas β-catenin levels increased by the administration of the above-mentioned Wnt agonist or neutralizing antibodies could improve healing^4, 7, 13, 15^. However, the role of the Wnt/β-catenin signaling pathway during the remodeling phase of bone fracture healing is currently unknown. Previous results have shown that the activation or inhibition of Wnt/β-catenin signaling alters and potentially influences the bone remodeling process, which might impair the speed and quality of bone fracture healing^12^. In addition, our previous studies indicated that constitutive activation of β-catenin impairs the terminal differentiation of osteoblasts^16, 17^. This effect might reduce the final bone fracture healing quality. Thus, we hypothesize that during the late stage of bone fracture healing, Wnt/β-catenin signaling should be maintained at an appropriate level to ensure better fracture healing quality. In the present study, β-catenin was constitutively activated, slightly activated or deleted in mice, and the effects of β-catenin on the bone remodeling phase and eventual bone fracture healing quality were investigated.

## Results

### Expression level of the genes associated with Wnt/β-catenin signaling in each group

**Figure 1:**
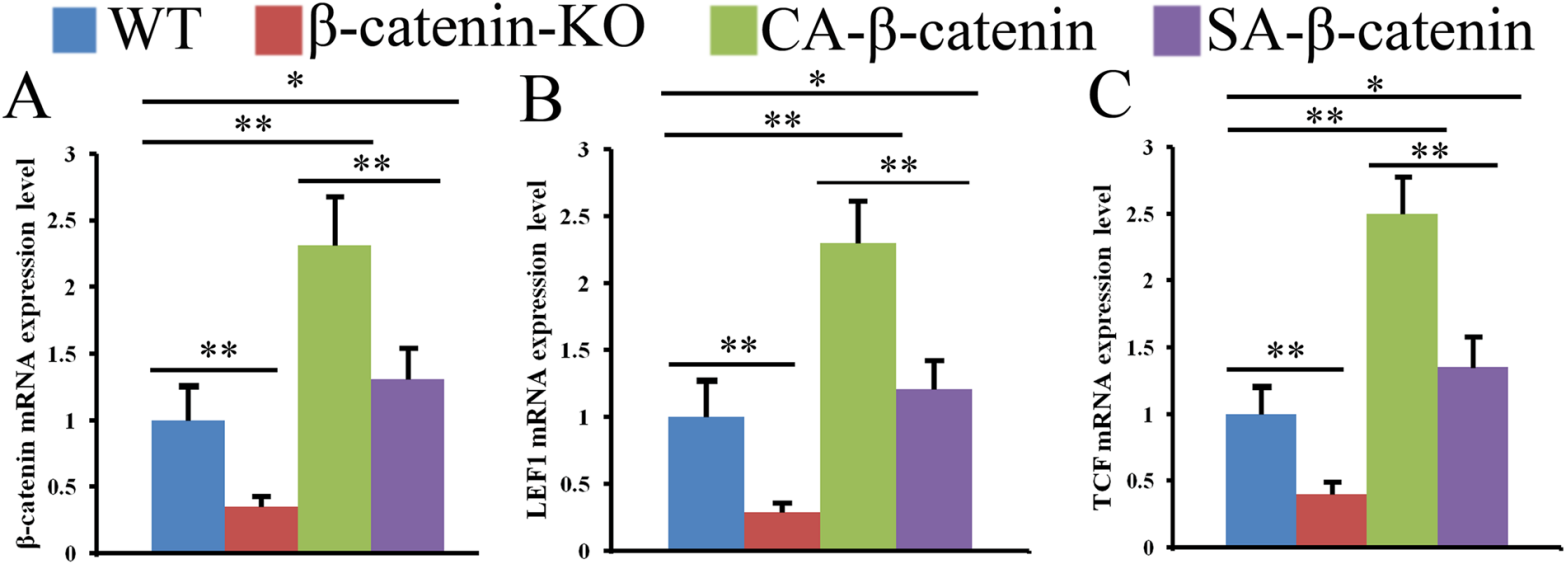
Wnt/β-catenin signaling pathway was deleted or activated in different levels at fracture sites in WT, β-catenin-KO, CA-β-catenin and SA-β-catenin mice. The mRNA expression level of β-catenin, TCF and LEF1 were examined by RT-PCR (**A**–**C**). The values are expressed as the means ± SDs. (n = 6/group, *p < 0.05, **p < 0.01).

The mRNA expression levels of β-catenin and its target genes in fracture sites after the injection of tamoxifen were examined by RT-PCR, and the results revealed that the β-catenin expression level in β-catenin-knockout (β-catenin-KO) mice were approximately 35.2% of that detected in wildtype (WT) mice, whereas the levels obtained after constitutive and slight activation of β-catenin (CA-β-catenin and SA-β-catenin) were 2.31- and 1.21-fold higher than that found in WT mice, respectively (Fig. 1A). Similar trends were found for the TCF/LEF1 mRNA expression levels in these groups (Fig. 1B and C).

### Characteristics of fracture healing in each group

**Figure 2:**
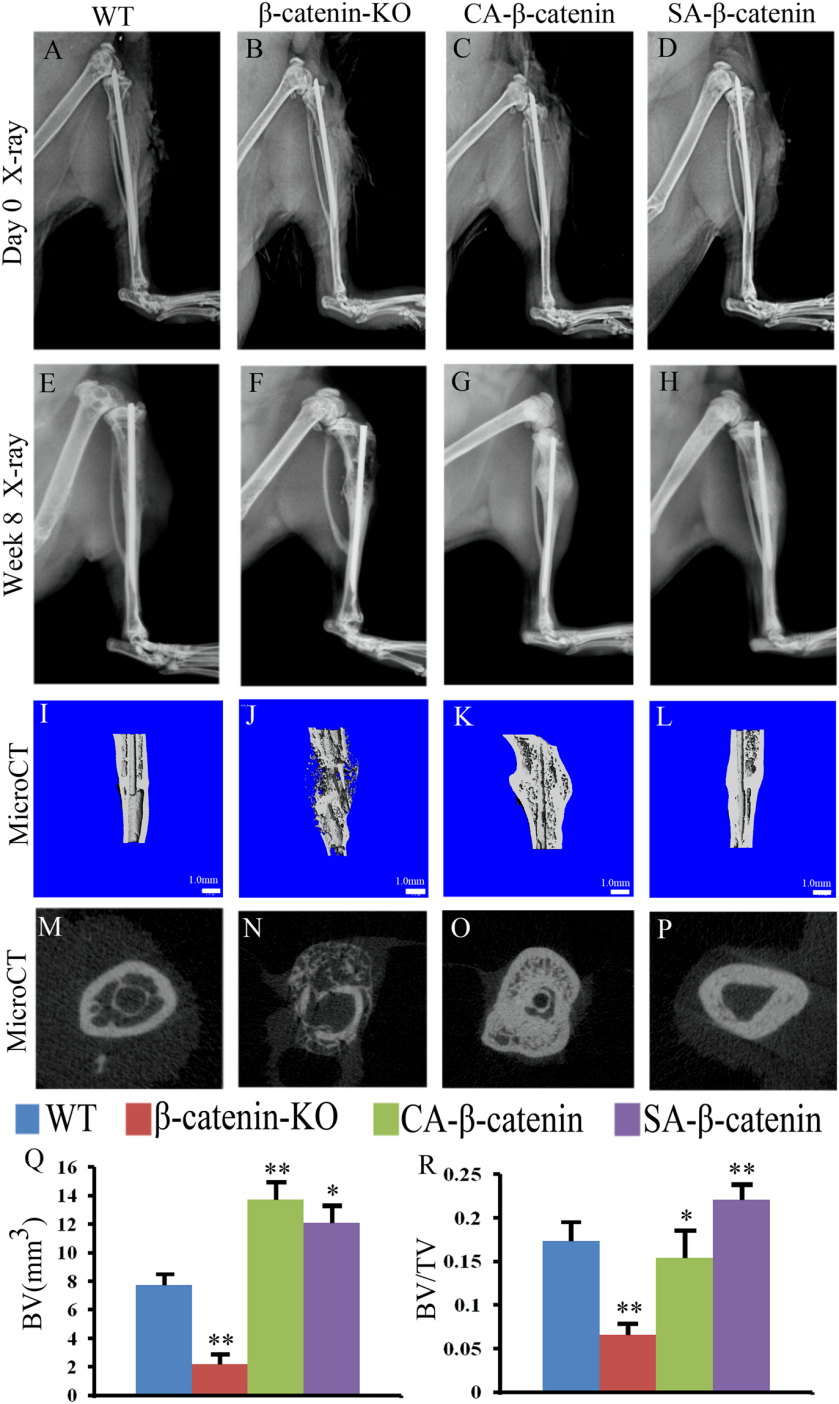
X-ray radiology and MicroCT examinations revealed that slight activation of β-catenin in the remodeling phases could improve healing quality. Representative X-ray radiographs of fracture sites in WT, β-catenin-KO, CA-β-catenin and SA-β-catenin mice at day 0 (**A**–**D**) and week 8 (**E**–**H**) post-fracture. (**I**–**L**) and (**M**–**P**) Representative 3-D and 2-D transverse reconstructive μCT-images in WT, β-catenin-KO, CA-β-catenin and SA-β-catenin mice at 8weeks post-fracture. (**Q** and **R**) Quantitative BV and BV/TV in WT, β-catenin-KO, CA-β-catenin and SA-β-catenin mice at week 8 post-fracture. The values are expressed as the means ± SDs. *p < 0.05, **p < 0.01 (Scale bar = 1 mm in **I–L**, n = 6/group).

The characteristics of fracture healing were assessed by X-ray, microCT and histology examinations. The radiographs of tibia collected immediately after surgery revealed proper alignment of the fracture at the start of the healing period in each group of mice (Fig. 2A–D). At eight weeks post-fracture, an X-ray examination revealed that a small volume of callus remained at the healed fracture site in both WT mice and SA-β-catenin mice (Fig. 2E and H), whereas the calluses were over-absorbed in β-catenin-KO mice (Fig. 2F), with radiographic densities lower than those observed in WT mice. In CA-β-catenin mice, a larger callus remained at the fracture site, reflecting a delayed remodeling process (Fig. 2G). These findings were further confirmed by microCT examination (Fig. 2I–P). The bone volume (BV) and tissue volume fraction (BV/TV) decreased significantly and the transverse sections of the microCT images showed decreased bone volume with a significant bone void in β-catenin-KO mice compared with WT mice (Fig. 2Q and R). The BV was increased significantly and BV/TV was decreased slightly in CA-β-catenin mice, whereas the values of BV and BV/TV were significantly increased in SA-β-catenin mice compared with WT mice (Fig. 2Q and R).

**Figure 3:**
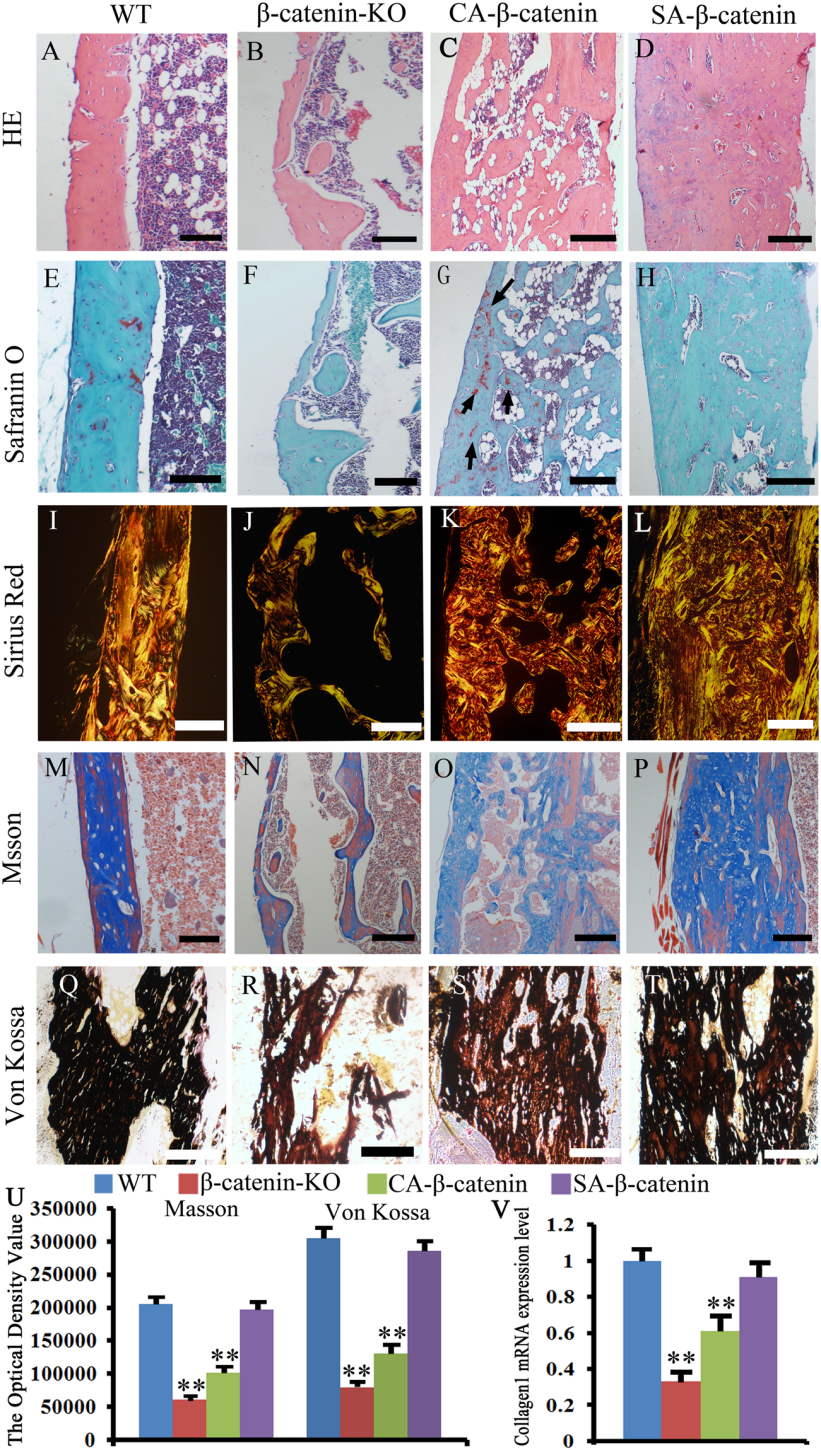
Histological examination revealed that SA-β-catenin mice have better histological morphology at week 8 post-fracture. (**A**–**D**) Representative HE staining of the fracture site in WT, β-catenin-KO, CA-β-catenin and SA-β-catenin mice. (**E**–**H**) Representative Fast Green/Safranin O staining images of the fracture site in each group. (**I**–**L**) Representative sirius red staining images of the fracture site in each group. (**M**–**P**) Representative Masson staining images of the fracture site in each group. (**Q**–**T**) Representative Von Kossa staining images of the fracture site in each group. (**U**) The optical density of Masson staining and Von Kossa staining images of the fracture site in each group. (**V**) mRNA expression level of collagen I at the fracture site in each group. The values are expressed as the means ± SDs. *p < 0.05, **p < 0.01. (arrows in **G**: the cartilage matrix, Scale bar = 100 µm in **A–H** and **M–P**, 50 µm in **I–L** and **Q–T**, n = 6/group).

Histologically, H&E and Fast Green/Safranin O staining revealed that the initial woven bone within the callus was remodeled into a lamellar structure in WT mice at week 8 post-fracture, achieving a morphology similar to that of non-fractured bone (Fig. 3A and E), whereas the callus was over-absorbed, with many bone absorption voids and no obvious formation of lamellar bone structure, in the fracture site of β-catenin-KO mice (Fig. 3B and F). In CA-β-catenin mice, H&E staining showed that most of the bone in the fracture site was woven bone and failed to remodel into lamellar bone (Fig. 3C). In addition, Fast Green/Safranin O staining revealed the presence of a small amount of residual cartilage matrix in the un-remodeled callus in CA-β-catenin mice (Fig. 3G). In contrast, nearly complete remodeling and no residual cartilage matrix were observed in SA-β-catenin mice (Fig. 3D and H).

Sirius red staining was employed to assess the collagen arrangement, and the results revealed that the collagen in β-catenin-KO and CA-β-catenin mice became disorganized compared with WT mice (Fig. 3J and K). In contrast, the collagen organization in SA-β-catenin mice did not significantly differ from that of the WT mice (Fig. 3I and L). Masson staining was employed to assess the collagen content, and the results showed that the collagen content decreased in β-catenin-KO and CA-β-catenin mice, but the collagen content in SA-β-catenin mice was almost the same as that in WT mice (Fig. 3M–P and U). Collagen 1 mRNA expression level tested by RT-PCR further confirmed these results (Fig. 3V). The mineralization level is an important indicator of the quality of fracture healing. Von Kossa staining revealed decreased mineralization levels in the β-catenin-KO and CA-β-catenin mice, whereas the mineralization levels of SA-β-catenin mice was almost the same as those in the WT mice (Fig. 3Q–U).

### Characteristics of bone remodeling parameters in each group

**Figure 4:**
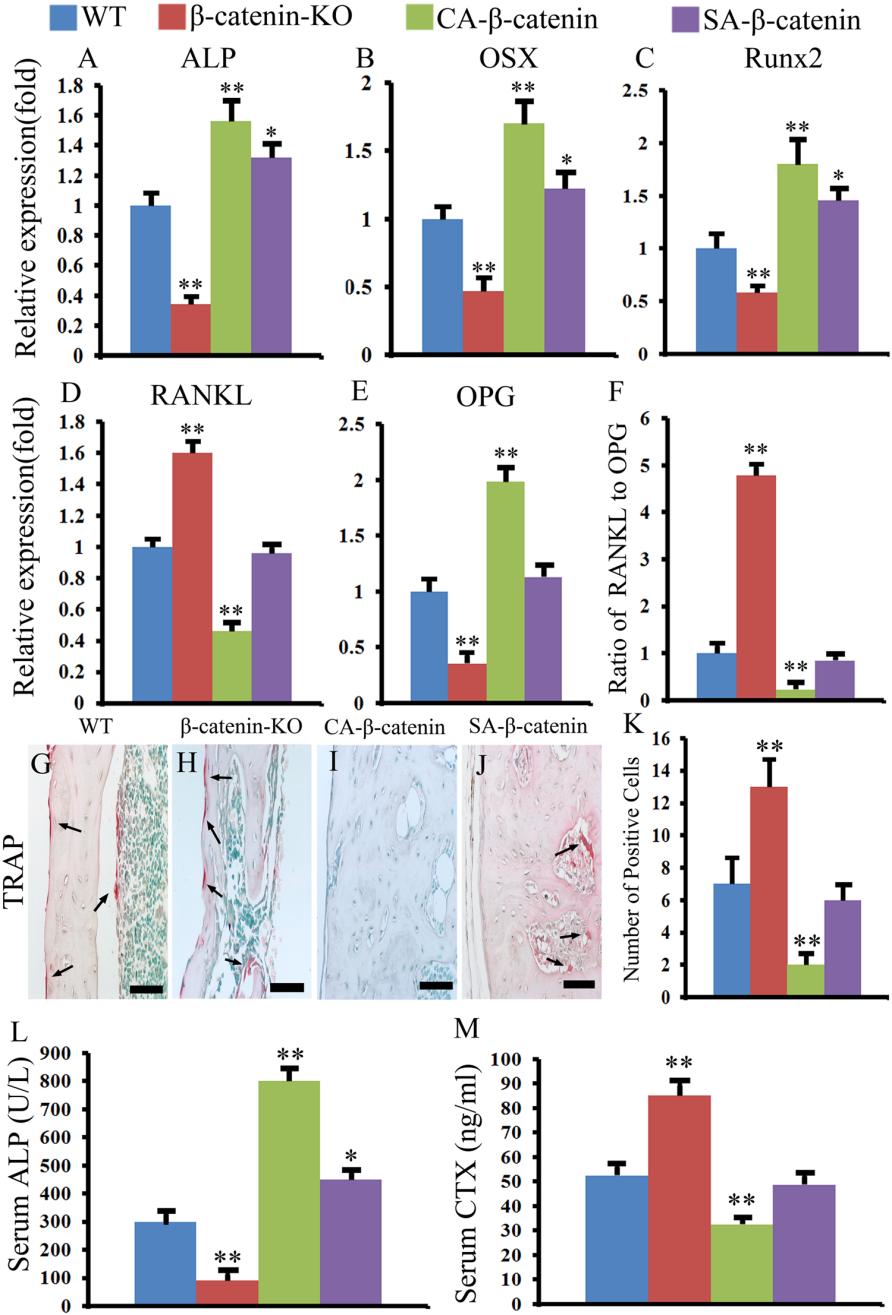
Slight activation of β-catenin in the remodeling phases enhanced bone formation without obvious hindering bone resorption. (**A**–**F**) The ALP, OSX, Rux2, RANKL, OPG mRNA expression levels and RANKL/OPG ratio in WT, β-catenin-KO, CA-β-catenin and SA-β-catenin mice. (**G**–**K**) Representative TRAP staining of tibia fracture calluses in each group and the quantitative data. (**L** and **M**) The serum concentration of ALP and CTX in each group. The values are expressed as the means ± SDs. *p < 0.05, **p < 0.01 (Scale bar = 25 µm in **G**–**J**, n = 6/group).

Bone remodeling is initiated by bone resorption, following by bone formation, and osteoblasts and osteoclasts are responsible for bone formation and bone resorption respectively. Alkaline phosphatase (ALP), Osterix (OSX) and Runt-related transcription factor 2 (Runx2) are useful markers of bone formation and osteoblasts differentiation. RT-PCR examinations revealed that the mRNA expression levels of these three genes was proportional to the level of β-catenin; specifically, the expression levels of these three genes were significantly decreased in β-catenin-KO mice, increased significantly in CA-β-catenin mice, and slightly increased in SA-β-catenin mice (Fig. 4A–C).

The level of receptor activator of nuclear factor-kappa B ligand (RANKL) and the RANKL/osteoprotegerin (OPG) ratio is important for osteoblast maturation. The expression level of RANKL and the RANKL/OPG were found to be increased significantly in β-catenin-KO mice, were decreased dramatically in CA-β-catenin mice, and showed no significant difference in SA-β-catenin mice compared with WT mice (Fig. 4D–F). Tartrate resistant acid phosphatase (TRAP) staining was then used to assess the number of osteoclasts, and the results revealed that the number of osteoclasts was significantly higher in β-catenin-KO mice and significantly lower in CA-β-catenin mice compared with the WT mice, whereas the number of osteoclasts in SA-β-catenin mice presented no significant differences compared with WT mice (Fig. 4G–K).

These findings were further confirmed by serum enzyme-linked immuno-sorbent assay (ELISA) testing. An ELISA analysis revealed an increase in the concentration of C-terminal telopeptides of type I (CTX), a marker of bone resorption, and a decrease in the concentration of ALP, a marker of bone formation, in β-catenin-KO mice compared with WT mice (Fig. 4L and M). In addition, a decreased concentration of CTX and an increased concentration of ALP were observed in CA-β-catenin mice, whereas an increased concentration of ALP and a slightly decreased concentration of CTX were observed in SA-β-catenin mice (Fig. 4L and M).

Taken together, the results revealed that the balance between bone resorption and bone formation differs among the groups, leading to different remodeling levels and contributing to differences in the bone fracture healing quality.

### Bone strength in each group

**Figure 5:**
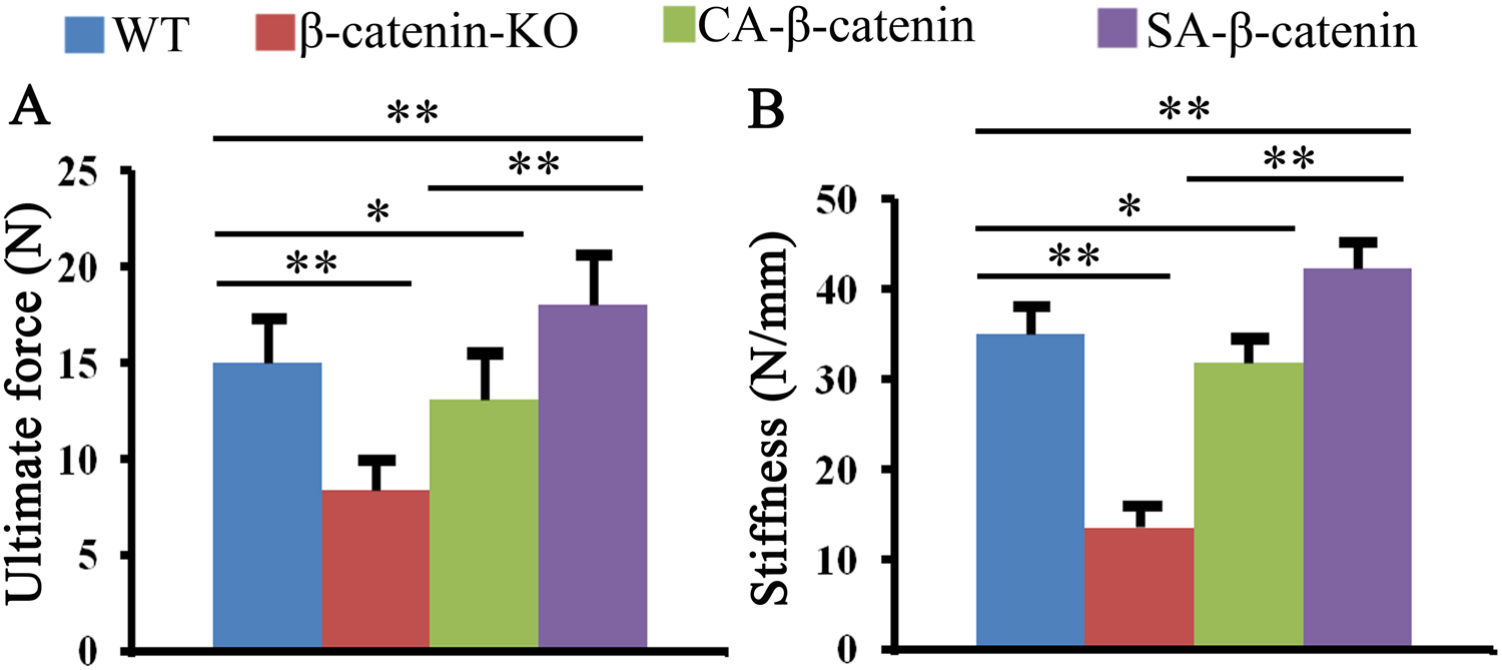
Mechanical test revealed that better bone fracture healing quality was obtained in SA-β-catenin mice. The ultimate load (**A**) and stiffness (**B**) of tibia fracture calluses in WT, β-catenin-KO, CA-β-catenin mice and SA-β-catenin mice. The values are expressed as the means ± SDs. *p < 0.05, **p < 0.01 (n = 6/group).

The bone strength was tested by a three-point bending test, which revealed that bone strength was decreased significantly in the fractured bones of β-catenin-KO mice, as shown by decreases in the ultimate load and stiffness. The bone strength in CA-β-catenin mice was greater than that in β-catenin-KO mice but lower than that in WT mice, and the bone strength in SA-β-catenin mice was greater than those in the other three groups (Fig. 5A and B).

## Discussion

Fracture healing can be divided into three overlapping phases (i.e., inflammatory, reparative and remodeling phases). Most studies have focused on the inflammatory and reparative phases, and little attention has been paid to the remodeling phase. The present study constitutes the first investigation of the role of Wnt/β-catenin signaling in the remodeling phase of bone fracture healing and revealed that Wnt/β-catenin signaling should be maintained at a proper level during the remodeling phase to ensure improved fracture healing quality.

The decrease in bone strength detected after β-catenin deletion is easy to understand: the β-catenin gene-deletion mice present decreases in the collagen content, the mineralization level and the bone mass, also disorder of the collagen structure. However, the decrease in bone strength observed in CA-β-catenin mice was not as readily predictable given the increase in callus mass^18–22^. Previous fracture studies of anti-resorptive agents revealed that although these agents delayed callus remodeling, the bone strength was generally enhanced due to increased callus mass. However, contrasting results were observed in CA-β-catenin mice compared with animals receiving anti-resorptive agent treatment. During bone remodeling, osteoblasts secrete osteoid, which is primarily composed of collagen type I. This bone matrix serves as the scaffolding for new bone. The matrix is then mineralized, and the collagen undergoes rearrangement. After mineralization, the osteoblasts either undergo apoptosis or terminally differentiate into osteocytes^23–26^. The constitutive activation of β-catenin during the remodeling phase of bone fracture healing results in the collagen disorganization and a decrease in the mineralization level. These findings were supported by the results obtained in our previous study and the studies conducted by Kim *et al*. and Minear *et al*., who demonstrated that constitutive activation of Wnt signaling results in decreases in the mineralization level due to increased cell proliferation and ultimately a delay in osteoblast differentiation^8, 9, 15, 17^.

Bone strength can be defined as the ability of bone to resist fractures, which is dependent on bone mass and quality^27^. The bone quality is controlled by the intrinsic properties of the bone material and structure. The material properties primarily refer to the degree of mineralization and collagen characteristics, and the structural properties mainly refer to the diameter and thickness of the cortices and trabecular bone mass. The constitutive activation of β-catenin during the remodeling phase results in several negative effects, including decreased mineralization levels and a disorganized collagen arrangement, and these effects might be greater than the positive effects of increased callus volume on bone strength, resulting in decreased bone strength. The treatment of animals with antiresorptive agents inhibits only bone resorption, with low or no impact on mineralization levels and collagen arrangement. Thus, bone quality was not disturbed in these animals, and their bone strength was generally enhanced due to an increased callus volume.

Interestingly, slight activation of β-catenin during the remodeling process increases bone strength relative to that of wildtype mice. In addition, there was a lack of changes in the mineralization and presence of well-organized collagen. These findings are consistent with the findings reported by Minear *et al*., who found that only transient activation of Wnt signaling by lipid vesicles containing Wnt could enhance bone fracture healing^9^.

One limitation of the current study was that serum concentraion of CTX and ALP were employed for assessing bone resorption and formation, respectively. Although these two markers are reported to be reliable, they are circulating markers and can only reflect a systemic level of bone resorption and formation. In addition, these markers could be influenced by many factors, such as the diet, metabolic state and urine output. Therefore, more accurate indexes should be identified to assess the local bone turnover state.

In summary, the present study constitutes the first investigation of Wnt/β-catenin signaling during the remodeling phase of bone fracture healing, and the findings suggest that Wnt/β-catenin signaling should be maintained at a proper level to ensure improved bone fracture remodeling and better bone quality.

## Methods

### Animal breeding, fracture model, and animal groups

#### Animal breeding

All animal procedures were approved by the Institutional Animal Care and Use Committee of Daping Hospital, Third Military Medical University, P. R. China. All methods were performed in accordance with approved guidelines of the Institute. Catnb^tm2Kem^ mice, Catnb^lox(ex3)^ mice and 3.2-kb mouse pro-collagen 1 promoter mice (3.2-kb Col1-Cre-ER^TM^) were obtained from Jianquan Feng (Department of Biomedical Sciences, Baylor College of Dentistry) and maintained on a C57BL/6 background. Catnb^tm2Kem^ mice possess loxP sites in introns 1 and 6 of the gene encoding β-catenin, resulting in a null allele when treated with Cre recombinase^28^. Catnb^lox(ex3)^ mice contain loxP sequences flanking exon 3, and Cre recombinase treatment of these animals results in the expression of a fully functional but stabilized β-catenin protein^16, 29^. Mice expressing tamoxifen-inducible Cre fusion protein, Cre-ER^TM^, under the control of the 3.2-kb mouse pro-collagen 1 promoter, which is active in osteoblasts, odontoblasts and tendon fibroblasts maintained on a C57BL/6 background were used to stabilize or delete β-catenin^29–31^. Tamoxifen (TM; Sigma-Aldrich, St. Louis, MO, USA) was injected to activate the function of the promoter^29^. Briefly, TM was dissolved in a small volume of ethanol and diluted with corn oil to a concentration of 10 mg/ml. The mice were intraperitoneally injected with TM at a dose of 75 mg/kg^29^. All of the animals used in the current study were male mice aged two months.

#### Animal groups and TM injection procedure

To constitutively activate β-catenin in the remodeling phase, TM was injected into Catnb^lox(ex3)^/3.2-kb Col1-Cre-ER^TM^ mice once every three days from weeks 3 to 8 post-fracture. To slightly activate β-catenin in the remodeling phase, TM was injected twice into Catnb^lox(ex3)^/3.2-kb Col1-Cre-ER^TM^ mice starting at week 3 post-fracture, and saline was then injected until week 8 post-fracture. To delete the gene encoding β-catenin in the remodeling phase, TM was injected into Catnb^tm2Kem^/3.2-kb Col1-Cre-ER^TM^ mice from week 3 to 8 post-fracture. Wildtype C57BL/6 mice were obtained from the animal center of Daping Hospital and used as control and were subjected to the same TM injection procedure as the CA-β-catenin mice. The efficiency of the TM dosing regimen to represent the β-catenin and TCF/LEF1 expression level was determined by real time-PCR, and the method is described in the following section.

#### Fracture model

Bone fractures were generated in eight-week-old male mice as described by Huang *et al*.^32^. Briefly, the mice were anesthetized through an intramuscular injection of pentobarbital sodium (0.05 mg/g, Chuangdong Co., Chongqing, China). Under rigorously aseptic conditions, the fur of the right tibia was shaved, and the skin sterilized with betadine solution prior to surgery. A small incision was made lateral to the patella, the patellar tendon was exposed, and a 27-gauge needle was used to ream a hole through the proximal tibial plateau and into the medullary cavity. The tibia was stabilized by inserting a sterile 0.25-mm-diameter stainless-steel pin through the reamed hole and down the tibia shaft. Following pin placement, a mid-diaphyseal fracture was created in the tibia using an electric saw. After fracture injury, the wound was irrigated with sterile saline and closed with sutures. Radiographs of the tibia were taken immediately post-operatively to ensure proper alignment of the fracture at the start of the healing period. Buprenorphine (Abbot Laboratories, Abbott Park, IL, USA) was administered in the drinking water for pain relief for the first three days after surgery. The animals were housed with free access to food and water and allowed unrestricted weight bearing after recovery from anesthesia.

### Radiographic imaging and tissue preparation

Eight weeks after surgery, the mice were deeply anesthetized, and radiographic images of their entire skeletons and the fractured tibia were obtained using a Faxitron MX-20 system (Faxitron, Wheeling, IL, USA). Subsequently, whole blood was extracted from the mice via a heart puncture, incubated at room temperature for 30 min and then centrifuged for 10 min at 5000 rpm. The serum was collected for the examination of bone biomarkers. The mice were then sacrificed by an overdose of the anesthesia, and the tibia were removed and fixed overnight. Some of the samples were either used for mechanical tests or directly embedded in plastic, and 8-μm undecalcified sections were cut for von Kossa staining. The remaining samples were decalcified in 10% ethylenediaminetetraacetic acid. Once they were adequately decalcified, the samples were tissue-processed, embedded in paraffin, and sectioned coronally at a thickness of 5 μm. The sections were de-paraffinized, rehydrated and used for hematoxylin and eosin (H&E), Fast Green/Safranin O, Sirius Red and Masson staining.

### Examination of serum bone biomarkers

The serum was used to determine the concentrations of CTX through an ELISA according to the instructions provided by the manufacturer. The ELISA kits were procured from Immunodiagnostic Systems, Ltd. (Boldon, UK). The concentrations of serum ALP were determined by routine methods using an automatic biochemical analyzer (UniCel DxC 800 Synchron Clinical Systems; Beckman Coulter, Fullerton, CA, USA).

### MicroCT examination

At week 8 post-fracture, the tibia were dissected and subjected to three-dimensional microCT analysis using a Viva CT 40 (Scanco Medical, Bassersdorf, Switzerland) following the procedural recommendations provided by the American Society for Bone and Mineral Research^33^. Ethanol was used as the scanning medium, the X-ray tube potential was 45 kVp, and the voxel size was 10 μm^3^. Images were reconstructed and analyzed with EVS Beam software using a global threshold of 1400 Hounsfield units. For analysis, the volume of interest (VOI) was defined as the region between the first and last slices with callus formation, resulting in an approximately 800-slice segment of bone. The total volume (TV), maximum moment of inertia (Imax), and bone volume fraction (BV/TV) were measured. Global thresholding was performed to distinguish between mineralized and non-mineralized tissue. Additionally, transverse sections of the central 1-mm region of the fracture were used to observe the bone remodeling process.

### Real-time polymerase chain reaction (real time-PCR)

Three mice from each group were sacrificed eight weeks after the operation. The whole tibia was dissected, and the whole fracture site was obtained. The samples were immediately shock-frozen in liquid nitrogen and were homogenized, and the total RNA was then isolated using the TRIzol reagent (Invitrogen) according to the manufacturer’s instructions. Real-time PCR using the SYBR green detection method was performed to examine the expression levels of β-catenin, TCF, LEF1, ALP, Osterix, Collagen1, Runx2, RANKL, and OPG. Glyceraldehyde-3-phosphate dehydrogenase (GAPDH) was used as a control, and the expression levels of specific genes are expressed as proportions relative to the mean GAPDH value. The primers that were used are presented in Table 1.

**Table 1:** Primers used for real-time PCR.

Genes	Primers Sequence (5′-3′)
β-catenin	F: 5′-ATGGAGCCGGACAGAAAAGC-3′
	R: 5′CTTGCCACTCAGGGAAGGA-3′
TCF	F: 5′-CCTCTCTGGCTTCTACTCCCT-3′
	R: 5′-CAGCCTGGGTATAGCTGCATGT-3′
LEF1	F: 5′-TGGCATCCCTCATCCAGCTATTGT-3′
	R: 5′-TGAGGCTTCACGTGCATTAGGTCA-3′
Collagen1	F: 5′-ATGTTCAGCTTTGTGGACCTCCGGC-3′
	R: 5′-GCCGGAGGTCCACAAAGCTGAACAT-3′
ALP	F: 5′-ACGAGATGCCACCAGAGG-3′
	R: 5′-AGTTCAGTGCGGTTCCAG-3′
OSX	F: 5′-CTCCTGCGACTGCCCTAA-3′
	R: 5′-ACATACCGTTCCGAAGCG-3′
Runx2	F: 5′-TGGTGCAGAGTTCAGGGAG-3′
	R: 5′-GAGGGACTTGAGACGTGGT-3′
RANKL	F: 5′-AACCAAGATGGCTTCTATTACC-3′
	R: 5′-AAGGGTTGGACACCTGAATG-3′
OPG	F: 5′-GCATTATGACCCAGAAACT-3′
	R: 5′-ACCTGAGAAGAACCCATC-3′
GAPDH	F: 5′-TCACTGCCACCCAGAAGA-3′
	R: 5′-AAGTCGCAGGAGACAACC-3′

### H&E staining

H&E staining was performed as described in a previous report^16, 17, 29^. Briefly, the sections were stained in Harris hematoxylin solution for 5 min, differentiated in 1% acid alcohol for 30 s, and bluing in 0.2% ammonia water for 30 s. Between each step, the sections were washed fully with tap water. The sections were subsequently rinsed in 95% alcohol, counterstained in eosin-phloxine solution for 30 s, rinsed in 95% alcohol for 2 min, in 100% alcohol for 3 min (twice), and in xylene for 2 min (twice), and coverslipped with Permount.

### Fast Green/Safranin O staining

The rehydrated sections were stained with Weigert’s iron hematoxylin for 1 min and rinsed in distilled water until clear. Subsequently, the sections were stained sequentially with 0.02% Fast Green for 5 min, 1% acetic acid for 30 s, and 0.1% Safranin O for 20 min. The slides were not rinsed between steps, and subsequently, the slides were rinsed with 95% alcohol for 2 min, 100% alcohol for 3 min (twice), and xylene for 2 min (twice) and coverslipped with Permount^29^. All chemicals were purchased from Chuandong Corporation (Chongqing, China). The purpose of these stains was to visualize stromal (hematoxylin in connective tissue), cartilaginous (Safranin O), and bony (Fast Green) tissues, allowing the reviewers to differentiate between these types of tissues for appropriate quantification.

### Sirius Red and Masson staining for collagen

Sirius Red staining was used to demonstrate differences in collagen structure as previously reported^17^. The prepared sections were stained with Weigert’s hematoxylin for 8 min, and the slides were then washed for 10 min in running tap water. Subsequently, the slides were stained with PicroSirius Red for 1 h and washed in acidified water. The water was removed, and the slides were dehydrated, rinsed in 95% alcohol for 2 min, 100% alcohol for 3 min (twice), xylene for 2 min (twice), and coverslipped with Permount. The collagen morphology (structure) was then observed under a polarized light microscope.

Masson staining was used to demonstrate the collagen content as previously reported^16^. Prepared sections were stained with Weigert’s iron hematoxylin working solution for 10 min, followed by Biebrich scarlet-acid fuchsin solution for 10 min. The sections were then differentiated in phosphomolybdic–phosphotungstic acid solution for 10 min and transferred directly (without rinsing) to aniline blue solution and stained for 5 min. After rinsing briefly in distilled water, they were differentiated in 1% acetic acid solution for 2 min, then rinsed in 95% alcohol for 2 min, 100% alcohol for 3 min (twice), xylene for 2 min (twice), and coverslipped with Permount. Collagen was stained blue in color. The content of collagen were assessed by analyzing the optical density with Image-Pro Plus 4.5 (Media Cybertics, The Netherlands).

### Von Kossa staining

Von Kossa staining was employed to monitor the mineralization ability of bone. After the sections were degreased and rehydrated, 100 µl of 2% silver nitrate solution was applied to each section, and the slides were exposed to strong light for 30 min. After the silver nitrate was removed, 5% sodium thiosulfate was added to the section for 10 s prior to rinsing with distilled water. The sections were then incubated with Van Gieson working solution for 5 min and prepared for observation^16, 17^. After von Kossa staining, the mineralized bone appeared black, with osteoid seams appearing bright pink and non-mineralized bone appearing pink. The optical density for the color of black was analyzed by using Image-Pro Plus 4.5 (Media Cybertics, The Netherlands).

### TRAP staining

TRAP staining was performed as described previously^16, 29^. Briefly, two Coplin jars (A and B) with 50 ml of stock basic incubation medium (9.2 g of sodium acetate anhydrous, 11.4 g of sodium tartrate dibasic dehydrate, and 2.8 ml of glacial acetic acid dissolved in 1000 ml of distilled water; the pH was adjusted to 4.7–5.0 with 5 M sodium hydroxide) were pre-heated to 37 °C. Fifty microliters of 2% naphthol AS-BI phosphate substrate in ethylene glycol monoethyl ether followed by the slides were added to jar A, and the jar was then incubated at 37 °C for 45 min. A few minutes prior to completion of the 45-min incubation, 1 ml of 5% pararosaniline chloride and 1 ml of 4% sodium nitrite were mixed for 30 s, and the resulting mixture was incubated at room temperature for 2 min without mixing, transferred into jar B and mixed well. The slides from jar A were then transferred to jar B without rinsing. Incubation at room temperature was performed for 1–3 min until color development, and the slides were then rinsed, counterstained with methyl green for 5 min, dehydrated, and covered with Permount. All chemicals were purchased from the Chuandong Corporation (Chongqing, China).

Pictures of each section were taken under a magnification of 400×, and the numbers of TRAP-positive cells corrected by bone area were counted in five random fields. The average numbers and standard deviations of TRAP-positive cells were calculated and subjected to statistical analyses.

### Mechanical testing

The fractured tibias, which had previously been measured by microCT, were tested for mechanical strength through a three-point bending test using a BOSE ElectroForce ELF 3200 computer-controlled testing machine, which has a force resolution of 0.05 N. During the test, each tibia was placed horizontally on two lower supports located 6.5 mm apart, with the anterior surface facing upward. The pressing force was applied vertically to the midshaft of the bone. Each bone was compressed at a speed of 0.05 mm/s until failure, and force-displacement data were collected every 0.01 s. Based on the data, a force-displacement curve was created, and the ultimate force (UF; N) was defined as the bending force at failure. The stiffness was calculated as the slope of the linear portion of the curve.

### Statistical analysis

All of the data are expressed as the means ± standard deviations. Statistical significance was evaluated through one-way analysis of variance (ANOVA) followed by Bonferroni’s post hoc test. The data were considered significant at *P* < 0.05.
